# Mexiletine-Induced Esophageal Ulceration: Two Case Reports and a Review of the Literature

**DOI:** 10.3390/reports8010009

**Published:** 2025-01-18

**Authors:** Matteo Ghisa, Ilenia Barbuscio, Erica Bonazzi, Matteo Fassan, Brigida Barberio, Marco Senzolo, Edoardo V. Savarino

**Affiliations:** 1 Department of Surgery, Oncology and Gastroenterology, University of Padua, 35124 Padua, Italy; matteoghisa@yahoo.it (M.G.); erica.bonazzi@unipd.it (E.B.); brigida.barberio@gmail.com (B.B.); marcosenzolo@hotmail.com (M.S.); 2 Unit of Gastroenterology, San Bortolo Hospital, Azienda ULSS 8 Berica, 36100 Vicenza, Italy; ilenia.barbuscio@gmail.com; 3 Dipartimento di Medicina, University of Padua, 35124 Padua, Italy; matteo.fassan@unpif.it

**Keywords:** mexiletine, esophageal ulcer, esophagitis, adverse event

## Abstract

**Background and Clinical Significance**: Mexiletine is a class 1B antiarrhythmic drug commonly prescribed for ventricular arrhythmias and neuropathic pain. It works as a blocker of the sodium channel that modulates cardiac conduction and reduces aberrant nerve signaling. While it is generally well tolerated, gastrointestinal side effects, such as nausea, vomiting, and abdominal pain, are relatively common. Esophagitis and esophageal ulcerations have been described as rare side effects; however, they are poorly documented in the literature. Esophageal ulceration induced by oral medications, termed pill esophagitis, occurs due to prolonged contact between the medication and the esophageal mucosa. Factors contributing to this phenomenon include improper administration, such as swallowing without sufficient water, taking medication before lying down, or inherent irritant properties of the drug itself. Mexiletine-induced esophageal ulceration has not been extensively reported, making such cases clinically significant and worth investigating. In particular, the prompt diagnosis of mexiletine-induced esophageal injury is essential for timely treatment initiation or the discontinuation of the drug, preventing complications such as bleeding, strictures, or perforation. Altogether, these actions are important to prevent the onset of potentially serious complications, such as bleeding, strictures, and the perforation of the esophagus. **Case Presentation**: Two different patients were included in this case report on mexiletine-induced esophageal ulceration: a 78-year-old woman affected by primary dilated cardiomyopathy and atrial fibrillation with high ventricular response and a 19-year-old man affected by dilated cardiomyopathy and systemic sclerosis. **Conclusions**: This case report underscores the importance of recognizing mexiletine-induced esophageal ulceration, and it advocates for timely diagnosis and management to optimize patient outcomes.

## 1. Introduction and Clinical Significance

Mexiletine hydrochloride is an orally active Class 1B antiarrhythmic agent, proven to be effective in the treatment of potentially lethal and drug-resistant ventricular arrhythmias [1,2,3,4].

While its safety profile is generally acceptable, gastrointestinal side effects, such as nausea, vomiting, and abdominal discomfort, are relatively common. However, severe adverse events like esophageal ulceration are rare and not well documented in the literature. The first time that this adverse event was described was in 1983, by Rudolph and colleagues [5]. Esophageal ulceration associated with medications, commonly termed “pill esophagitis”, arises when a drug remains in prolonged contact with the esophageal mucosa, causing localized irritation or direct injury [2]. Factors such as swallowing pills without adequate fluid, taking medications before lying down, or intrinsic corrosive properties of the drug increase the risk of esophageal damage. Medications like tetracyclines, bisphosphonates, and NSAIDs are well-known culprits due to their chemical properties and the way they interact with the esophageal mucosa [6,7,8,9,10]. Indeed, due to their acidic nature, these molecules can cause esophageal irritation and damage the esophageal lining if they remain in prolonged contact with the esophagus, especially when swallowed without enough water or if the patient lies down soon after ingestion.

However, mexiletine-induced esophageal ulceration is rarely reported, highlighting the need for increased awareness and documentation. The clinical significance of mexiletine-induced esophageal ulceration lies in its potential to cause severe complications if unrecognized or untreated. Esophageal ulceration can lead to debilitating outcomes such as strictures, bleeding, or even perforation, which significantly impact a patient’s quality of life and require urgent medical intervention. Diagnosing this condition is challenging, as its symptoms often mimic common disorders like GERD or infectious esophagitis, potentially leading to delays in appropriate care. Furthermore, such adverse effects may necessitate discontinuing mexiletine, depriving patients of its critical benefits in managing ventricular arrhythmias or neuropathic pain. This case series highlights the importance of clinician awareness regarding this rare complication, enabling timely diagnosis and prevention strategies, such as educating patients on proper administration techniques. Documenting these cases not only expands the medical knowledge base but also aids in improving patient safety and treatment outcomes. This article is a revised and expanded version of a paper entitled “MEXILETINE INDUCED ESOPHAGEAL ULCERATION: TWO CASE REPORTS” [11], which was presented as an abstract at the 26th National Congress of Digestive and Liver Disease.

## 2. Case Presentation

### 2.1. Case Report #1

**Figure 1:**
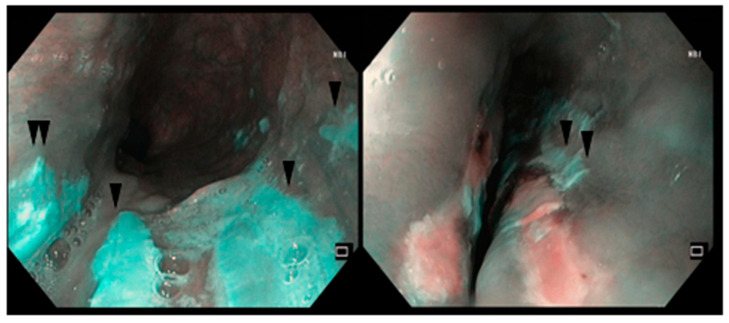
EGD images with NBI showing linear ulcerations at the distal esophagus, 6 cm in length, covered by fibrin from the patient of case report #1. The blue color is due to the use of virtual chromoendoscopy.

A 78-year-old woman, affected by primary dilated cardiomyopathy and atrial fibrillation with high ventricular response, was admitted to a cardiology unit for recurrent syncope. She underwent echocardiography, showing severe atrial and ventricular dilation, with reduced pump function, diffuse hypokinesia, and severe mitral and tricuspid insufficiency. At admission, the patient had been taking mexiletine for six months. During the hospitalization, for the persistence of dyspepsia, abdominal bloating, and regurgitation, esophagogastroduodenoscopy (EGD) was performed, and Figure 1 shows the linear ulcerations at the distal esophagus, 6 cm in length, covered by fibrin.

Biopsy samples were taken and histological examination revealed marked parakeratosis and the detachment of the superficial layers of the lining epithelium, associated with mild lymphocytic and granulocytic infiltrates, compatible with “sloughing esophagitis” (esophagitis dissecans superficialis). Based on the histological findings and clinical history, suspicion of an iatrogenic origin of the esophagopathy was posed; thus, mexiletine was stopped and proton pump inhibitors (PPIs) b.i.d., as well as sucralfate, were started. Gastroesophageal symptoms gradually disappeared and follow-up EGD two months later showed the resolution of ulcerations.

### 2.2. Case Report #2

**Figure 2:**
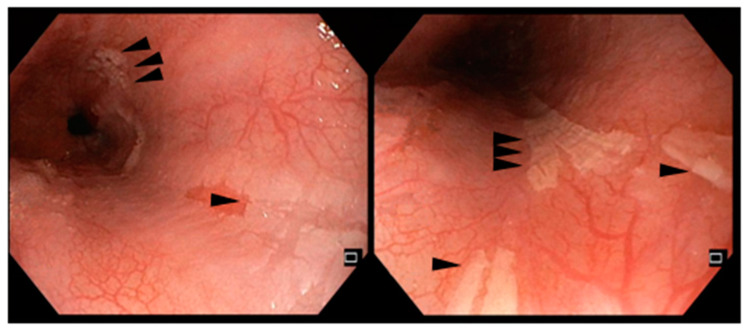
Multiple discontinuous distal esophageal ulcerations about 15 cm long, with fibrinous membranes, from the patient of case report #2.

**Figure 3:**
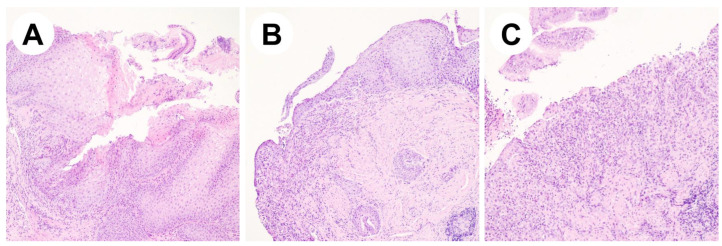
Representative histological pictures of case report #2. (**A**) Hyperplasia of the proliferative compartment and high-grade parakeratosis. (**B**,**C**) Ulceration of the mucosa associated with inflammatory granulation tissue and infiltration by leukocytes of the lamina propria and of the muscularis mucosae. (H&E stain; original magnifications 10× and 20×).

In a 19-year-old man, ventricular arrhythmias were discovered during a routine sports medical examination. He underwent further investigations, finding that he was affected by dilated cardiomyopathy and systemic sclerosis. Immunosuppressive drugs were then administered in association with antiarrhythmic therapy with mexiletine and implantable cardioverter defibrillator placement. For the severity of the autoimmune myocarditis and the patient’s young age, assessments for heart transplantation were started. Amongst them, EGD showed esophageal ulcerations with the presence of fibrinous membranes 15 cm lengthwise in the middle and distal esophagus, as reported in Figure 2. Histological examination displayed inflammatory granulation tissue with the granulo-lympho-monocytic infiltration of the lamina propria associated with hyperplasia of the proliferative compartment and high-grade parakeratosis. Given the endoscopic and histological findings (Figure 3), even in the absence of symptoms, PPI therapy was started, and treatment with sucralfate and hyaluronic acid was started. Mexiletine was not discontinued due to the severity of the cardiomyopathy. EGD performed two months later showed a remission of the ulcerations, but with persistence of mild esophagitis.

## 3. Discussion

Many cases of pill-induced esophagitis are reported in the medical literature associated with a large variety of drugs. We performed a review of the literature regarding mexiletine-related esophageal involvement, including one clinical trial, one case series, and four case reports published from 1981 until May 2019. Only seven articles directly cited the presence of ulcers or esophagitis as a mexiletine-related adverse event, mostly as single case reports, dated back about thirty years ago [5,12,13,14,15].

Nevertheless, papers concerning mexiletine safety described gastrointestinal adverse reactions such as inflammation, bleeding, and ulceration and were reported in less than 0.1% [16]. Another study including 100 patients reported esophageal spasms in 3% of them, which can be an additional risk factor for direct drug-induced esophageal damage [17]. Moving to single case reports, patients described in the literature and their characteristics are resumed in the table below (Table 1).

Regarding possible pathogenic mechanisms underlying esophageal damage, the most probable hypothesis is of mexiletine-induced esophagitis by prolonged direct contact between the mexiletine capsule and the esophageal mucosa surface. Other mechanisms, such as elevated topical hyperosmolality, local caustic injury, or the disruption of the normal cytoprotective prostaglandin barrier, responsible for esophageal damages in other medications (e.g., potassium chloride, tetracycline, and NSAIDs), are probably not implicated, given the chemical nature of mexiletine.

Mexiletine-related esophageal damage is also associated with the modality of the administration of the pill, i.e., the position of the patient when taking the pill and the quantity of water swallowed together with it. In detail, the intake of the drug in clinostatism and with little amount of water significantly slows esophageal transit [18]. Furthermore, the ingestion of the capsule immediately prior to sleep, given both the lying position and the reduced salivation and swallowing frequency, increases the risk of mexiletine-induced esophagitis.

Esophageal anatomy may play a pathogenic role in pill-induced esophagitis. The areas most commonly affected are those where the esophageal lumen is anatomically narrower. This includes regions compressed by the aortic arch or an enlarged left atrium, as well as areas with esophageal rings, stenosis, or diverticula. Additionally, the esophagogastric junction is physiologically susceptible. The gelatin capsule coating of mexiletine, being sticky, increases the likelihood of esophageal retention. Advanced age is another risk factor, though prolonged bed rest, xerostomia, cardiac dilation, and multidrug therapy may introduce bias in assessing this association. In turn, mexiletine itself could cause esophageal dysmotility. A study conducted in rabbits showed that it can inhibit lower esophageal sphincter relaxation in a dose-related manner [19]. Mexiletine-induced esophageal ulceration is a rare but challenging condition to diagnose and manage due to its nonspecific presentation, often mimicking other gastrointestinal disorders such as gastroesophageal reflux disease or peptic ulcers. Symptoms like dysphagia or retrosternal pain are often attributed to underlying cardiac conditions, delaying diagnosis. Limited awareness and sparse case reports further complicate timely identification. Management involves the discontinuation of the drug and supportive measures, but delayed diagnosis can lead to significant morbidity. Early recognition is crucial to prevent complications and improve outcomes.

Considering our two patients, the above-mentioned risk factors could be involved in the pathogenesis of esophageal manifestations. The first patient was enticed and the second one could have esophageal motility disorders due to scleroderma; although, in the latter hypothesis, the resolution of the ulcerations after drug cessation, the mid-esophagus location of the lesions and the lack of symptoms reported (i.e., dysphagia or typical reflux symptoms) seem to suggest that there was no esophageal involvement of scleroderma and that GERD was not the cause of the mucosal injuries. Moreover, particularly in the first case, the histology was clearly suggestive of drug-induced esophagitis.

Concerning therapeutic management, the discontinuation of mexiletine should be recommended and, if not possible, the intake of the capsule in an upright position and with an adequate amount of water is suggested. At the same time, PPIs and sucralfate administration are indicated for symptom resolution and mucosal healing [20]. New agents such as hyaluronic acid and chondroitin sulphate could be promising, but they still need further evaluation [21].

## 4. Conclusions

In conclusion, recognizing mexiletine-induced esophageal ulceration is critical for ensuring prompt diagnosis and management to prevent severe complications. Clinicians should be aware of this rare adverse effect, particularly in patients presenting with new-onset dysphagia or chest pain while on mexiletine therapy. Early identification and the cessation of the offending drug, along with appropriate treatment, can prevent severe complications. These cases underscore the importance of vigilance in monitoring for uncommon drug-related adverse events and highlight the need for comprehensive patient education regarding potential side effects to facilitate timely reporting and management.

## Figures and Tables

**Table 1:** Characteristics of patients described in the literature, affected with mexiletine-induced esophageal ulcerations.

	Patient 1	Patient 2	Patient 3	Patient 4	Patient 5
**Age (yo)**	72	63	77	73	67
**Sex**	woman	man	woman	man	woman
**Duration of mexiletine treatment**	single administration	3 months	long time (not specified)	1 month	long time (not specified)
**Symptoms**	severe epigastric pain	hemoptysis	asymptomatic	dysphagia for 2 months	epigastric pain and dysphagia
**Endoscopy**	flat ulcer on the posterior wall of the esophagus with a lilac-pink surface	long ulcer with edematous contours in the right piriform sinus	two 5 mm longer and reddish kissing ulcers, at the cervical esophagus	two 1–1.5 cm large ulcers at the middle esophagus	vertical white striped mass covering the entire surface of the esophagus
**Histology**	diffuse purulent esophagitis, without sign of malignancy	not performed	not performed	exclusion of malignancy or infection	spongiosis of the middle layer of squamous epithelium
**Therapeutic modification**	suspension of mexiletine therapy with chamomile, antacids, and cimetidine	mexiletine suspension	changing modality of assumption of mexiletine (with water and upright)	reduction in mexiletine dosage, starting ranitidine, and suspension of Ascriptin	mexiletine suspension
**Outcome**	symptoms resolution and ulcer healing after 15 days	ulcer healing after 7 days	quick resolution of symptoms. EGD not performed	quick resolution of symptoms. EGD not performed	quick resolution of symptoms. Endoscopic resolution after 4 weeks
**Reference**	[5] Rudolph et al., 1983 (German)	[5] Rudolph et al., 1983 (German)	[13] Penalba, 1986 (French)	[12] Adler et al., 1990 (English)	[14] Sakai et al., 1992 (Japanese)

## Data Availability

The original data presented in this study are available on reasonable request from the corresponding author. The data are not publicly available due to privacy.
